# Introduction to special issue on “Responsive Materials and Systems: Toward Smart and Precision Medications”

**DOI:** 10.1002/btm2.10045

**Published:** 2016-11-11

**Authors:** Zhen Gu

**Affiliations:** ^1^ University of North Carolina at Chapel Hill and North Carolina State University; ^2^ Molecular Pharmaceutics Division Eshelman School of Pharmacy University of North Carolina at Chapel Hill; ^3^ Dept. of Medicine University of North Carolina School of Medicine

Spurred by advances in materials chemistry, molecular pharmaceutics and micro/nanobiotechnology, stimuli‐responsive “smart” materials and systems have been studied extensively for various applications, including drug delivery, diagnosis, tissue engineering, and biomedical devices. In drug delivery, the dosage‐, spatial‐ and/or temporal‐ controlled release of therapeutics significantly enhances the treatment efficacy in a precise manner. The development of stimuli‐responsive materials also allows noninvasive or minimally invasive monitoring in real‐time for achieving next‐generation diagnostics. In addition, the smart systems with the capability of communicating and interacting with cells are highly desirable for engineering regenerative medications and biomedical devices.

This theme issue focuses on the responsive materials and systems for a range of biomedical applications, with a collection of ten relevant research or review papers. For diabetes treatment, Mitragotri and coworkers^1^ describe a novel mucoadhesive intestinal device entrapped in a capsule with a pH‐responsive enteric coating for oral delivery of insulin. This device loaded with insulin can effectively decrease blood glucose levels of diabetic rats. Gu and coworkers^2^ summarize a variety of stimuli‐responsive delivery systems for diabetes treatment, including the pH‐sensitive materials for oral delivery, the glucose‐responsive delivery systems, and the on‐demand delivery approaches by external ultrasound or light. For cancer therapy, Tong and Feng^3^ discuss general principles in polymer‐drug conjugate design such as the synthetic strategies, the choice of the responsive linkers between the drug and polymer, and the in vivo delivery barriers. Liu and Liang^4^ review reactive oxygen species (ROS)‐responsive system as well as multiple stimuli systems for enhanced delivery of therapeutics. For tissue engineering, Hammond and collaborators^5^ develop nanoscale polyelectrolyte complexes to deliver insulin‐like growth factor‐1 (IGF‐1) for cartilage repair. They demonstrate IGF‐1 is released by nanocomplexes within the joint space over four weeks, protecting cartilage from degradation and mitigating joint inflammation.

Remote utilization of physical triggers, such as light, magnetic force and temperature to achieve spatiotemporal activation is an emerging research topic in this field. Lovell and Miranda^6^ discuss different mechanisms of the light‐induced liposome permeabilization for controlled drug delivery, including light‐induced oxidation, photocrosslinking, photoisomerization, photocleavage, and photothermal release. Xu and coworkers^7^ describe the development of near‐infrared light‐responsive liposomes for enhanced gene transfection through the photothermal effect. Gaharwar and coworkers^8^ review the applications of smart hydrogels based on magnetic nanoparticles and thermoresponsive polymers in therapeutic drug delivery, bioimaging, and regenerative medicine.

In the remainder of this issue, Webber^9^ focuses on the preparation of responsive self‐assembled materials for biomedical applications, including engineering therapeutics and devices for biological sensing and disease diagnostics; Cui and coworkers^10^ review peptide‐based supramolecular nanostructures and hydrogels for biological stimuli‐triggered delivery of biologics.

Collectively, this issue highlights the exciting research work and key advances in leveraging responsive materials for smart and precision medications, the clinical translation of which would revolutionize health care, profoundly enhancing patients’ health and improving their quality of life.

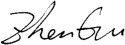

Zhen Gu^1,2,3^

^1^University of North Carolina at Chapel Hill and North Carolina, State University

^2^Molecular Pharmaceutics Division, Eshelman School of Pharmacy University of North Carolina at Chapel Hill

^3^Dept. of Medicine University of North Carolina School of Medicine
